# Myopia: The Evidence for Environmental Factors

**DOI:** 10.1289/ehp.122-A12

**Published:** 2014-01-01

**Authors:** Tim Lougheed

**Affiliations:** **Tim Lougheed** has worked as a freelance writer in Ottawa, Canada, since 1991. A past president of the Canadian Science Writers’ Association, he covers a broad range of topics in science, technology, medicine, and education.

Centuries ago, dedicated monastic scribes or cloistered seamstresses might have blamed failing eyesight on their particular type of near-focus “close work.” By the late twentieth century, such blame was expanded to include “close leisure,” such as countless hours spent sitting in front of the television, and most recently squinting at high-resolution monitors on everything from gaming consoles to cell phones.

However, despite ongoing attempts to tie these close behaviors to the onset of nearsightedness, or myopia, researchers have not come up with convincing results. On the other hand, a rapidly growing body of research on populations in Asia is yielding strong evidence linking diminishing levels of exposure to outdoor light with a prevalence of myopia that is approaching epidemic proportions.^1^^,^^2^^,^^3^

“Keep myopia at bay,” chirp colorful screensavers and wallpapers offered by Singapore’s Health Promotion Board. “Go outdoors and play.” The irony of flashing messages on a computer monitor to get children away from that same monitor is not lost on the researchers who have been charting the rise of chronic nearsightedness in the region’s populations.

Singapore’s health authorities launched the public awareness campaign in response to a sharp spike in myopia rates among children and young people that was flagged by the country’s military. Military service is compulsory in Singapore, and the eye examinations conducted on incoming conscripts effectively provide a population-wide survey. By the late 1990s, these examinations pointed to a prevalence approaching 80%.^4^ Comparable findings have been reported in other Asian countries such as Taiwan^5^ and South Korea.^6^

Meanwhile, many parts of Asia have undergone rapid economic development, and demanding educational regimes have been implemented over the past two decades to create a highly skilled, dedicated workforce to keep building the momentum of this economic drive.^7^^,^^8^ Not only do children spend most daylight hours in classrooms, they remain equally sequestered at home, either finishing up schoolwork or engaged in leisure activities that keep them glued to one screen or another.^7^^,^^8^^,^^9^

“It took me a while to realize how distorted a child’s experience of the world is in China,” says Ian Morgan, an Australian ophthalmology researcher who spends about five months of each year in Guangzhou studying myopia. “In Guangzhou, the climate is hot and sticky for most of the year, and people get outdoors in the evening. But you do not see children of school age, because they are at home doing their homework,” he says.

This lifestyle appears to exact a toll on young eyes. Population surveys in Guangzhou, Singapore, and Taiwan point to these areas as hot spots for children becoming myopic earlier, with more of them being more severely myopic.^4^^,^^5^^,^^10^^,^^11^ But even as the precise factors responsible for the increase are still being debated and explored, a mounting body of evidence cites time spent outdoors as crucial to the healthy development of the eye.

Although myopia is becoming more prevalent worldwide, some areas are more affected than others. Surveys in the United States and most especially in Australia, for example, yield prevalence figures for the general population that are consistently much lower than comparable figures in Southeast Asia.^12^^,^^13^

The sharp region-specific disparity also appears to affect primarily the younger generation. Among adult populations around the world, prevalence rates show less variation across countries (although future surveys are expected to show similar disparities in adult rates as today’s youth age). Research drawing on data from the 1999–2004 iterations of the National Health and Nutrition Examination Survey (NHANES) estimates that U.S. myopia prevalence can range from 33.1% across the entire adult population to as little as 25.1% for Mexican Americans.^13^ By comparison, one study of myopia in Singaporean Chinese, Malay, and Indian adults reported rates of 38.7%, 26.2%, and 28.0%, respectively.^12^

Myopia may not be as grave a health problem as cancer or heart disease, but there is more at stake than a population saddled with the expense and inconvenience of coping with glasses or contact lenses. Studies suggest the condition may be a risk factor for more serious eye problems including cataract^14^ and glaucoma,^15^ although myopia’s relationship to these other problems is still unclear. (On the other hand, myopia is negatively associated with age-related macular degeneration.^16^) Young people afflicted with the most serious degree of myopia display few other symptoms, but middle-aged individuals with “high” (severe) myopia exhibit many pathologies of the eye.^8^

## A Basis in Genetics?

Myopia stems from what amounts to a small though apparently important physical deformity: a lengthening of the eyeball along its horizontal axis. Myopia comes in varying degrees, depending on the extent of this distortion. According to Morgan, a 1-mm increase in the length in a 25-mm eye leads to moderate myopia, where objects more than 1 m away appear blurry. A 2-mm increase puts the individual in the range of high myopia, in which objects become blurred beyond 16 cm.

“It’s very simple,” explains Donald Mutti, a member of faculty at the College of Optometry at The Ohio State University. “A myopic eye is an eye that’s kind of too big for its britches. It’s too large, and we just have to get it to slow down a little bit, without influencing function. If we really understand the physiology of eye growth, there are probably many opportunities to influence the chain of events that control growth and how big the eye eventually gets.”

In Singapore and several neighboring countries, the prospect of achieving such an understanding has become more than just a tantalizing intellectual milestone; it would set the stage for intervention strategies to tackle the causes of myopia rather than simply correct its symptoms. A great deal of the research literature in this field therefore originates in Asia, where public discussion of myopia, along with its social and economic implications, overshadows any talk of the issue in the Western world.

At the heart of this scientific enterprise is the distinction between nature and nurture. Variations in myopia prevalence among different ethnicities suggest a genetic contribution of some sort. Clues to this potential contribution have come from studies such as the Collaborative Longitudinal Evaluation of Ethnicity and Refractive Error (CLEERE), which included more than 4,000 U.S. children from four ethnic groups. Asian and Hispanic participants were found to have higher prevalence of myopia (18.5% and 13.2%, respectively) than black and white children (6.6% and 4.4%, respectively).^17^

For Mutti, who was part of the CLEERE Study Group, such data can suggest genetic links to myopia but say little about the nature of those links and how they determine prevalence. He recommends careful scrutiny of any figures related to changes in prevalence, which might be exaggerated by samples from specific populations that are being influenced by other factors. Indeed, the four different ethnic groups in CLEERE came from four different locations in three states; thus, differences attributed to ethnicity could easily have come from environmental factors.

The U.S. National Eye Institute (NEI), a branch of the National Institutes of Health, conducted one of the most sophisticated attempts to date to assess myopia prevalence in the United States. Susan Vitale, an NEI epidemiologist who is also an adjunct associate professor of ophthalmology at the Johns Hopkins University School of Medicine, led the comparison of NHANES data from the early 1970s and the early 2000s.

NHANES captures nationally representative samples of the U.S. population each time it is conducted. The ethnic make-up of that population has shifted over the last three decades; Asian and Hispanic representation was much more limited in the earlier versus later surveys, so data from these population groups were not included in the comparison.

To complicate matters, the methodology for measuring myopia also changed over the years. In the 1970s, detailed measurements were taken only on participants with more severe refractive error, and those were made through retinoscopy, a manual observation of how different strengths of corrective lenses affect the reflections bounced off the retina. By the time the second survey began in 1999, all participants were assessed with an autorefractor, a computer-controlled system that measures how well the eye can focus images on the retina.

More than 5,000 people participated in NHANES I, conducted in 1971–1972, with the vast majority classified as black or white; almost 10,000 people in the 1999–2004 survey fell into these two categories. Some striking increases in the prevalence of myopia emerged. In the 1970s, the rate was only 12% among black participants aged 12–17, but by 2004 that figure was 31.2%. Similarly, the rate among white participants in the same age group moved from 25.8% to 34.5%. The rates for participants in older age groups increased even more, so that the overall average reached 33.5% among black participants and 43.0% among white participants.^18^

These numbers are well below similar surveys in Asia, but Vitale says, “Given this evidence, and putting it together with the kinds of findings people were seeing in other countries, it seemed pretty clear that the prevalence had indeed increased.”

For Mutti, that increase raises questions about the sampling and measurement that went into the data. He praises the quality of the NHANES data and the NEI’s analytical approach, but he maintains that the distinct character of data sets spanning three decades makes it difficult to arrive at accurate figures. “I would accept that the prevalence of myopia is not a fixed quantity,” he says. “Reports from Asia suggest that there might be increases. There could be increases in the United States, but my bottom line sentiment is that the increases aren’t quite as severe—if they’re occurring—as publicized.”

## Environmental Factors

Less controversial is the evidence suggesting that time outdoors protects against the development of myopia.^2^^,^^19^^,^^20^^,^^21^^,^^22^ “Most ophthalmologists and optometrists were taught that myopia was a [strictly] genetic disorder, but the evidence has stacked up solidly against this idea,” Morgan says. He points to the ambitious work of the Consortium of Refractive Error and Myopia, which carried out a meta-analysis on more than 55,000 individuals from four continents, but still fell short of finding a firm genetic link.^23^

Morgan’s work, instead, has focused on the role of ambient light on development of myopia, specifically the impact of time spent outdoors.^24^ He and his colleagues proposed that any protective effect of time outdoors was most likely to be mediated by bright light stimulating the release of a transmitter dopamine from the retina; light is known to stimulate dopamine release, and drugs that mimic the effects of dopamine reduce eye growth. He led the seminal Sydney Myopia Study, which developed a comprehensive questionnaire to pinpoint exactly how much time children spend indoors and outdoors, and what types of activities they do in both settings.

Morgan says his hypothesis is supported by work showing that experimental animals, raised under conditions that normally lead to myopia but with the addition of bright lights, did not become myopic.^25^^,^^26^ In addition, drugs that block dopamine have also been shown to block the protective effect of light.^27^

Seang-Mei Saw, an ophthalmic researcher with the National University of Singapore, led a key comparison of 6- and 7-year-old children of Chinese ethnicity living in Sydney and Singapore.^28^ Although the two groups presumably shared a similar genetic predisposition to myopia, their measured prevalence of this condition contrasted sharply: 3.3% among those living in Sydney versus 29.1% for those in Singapore.^28^ A comparison of the children’s lifestyles further revealed that the Sydney group engaged in just as much, if not more, near work than their Singapore counterparts.

Saw recalls that these findings represented a turning point in the perception of environmental influences on myopia. While she and her colleagues were actively seeking near-work conditions that might have an effect on eye development, the researchers were surprised by the glaring point of contrast between the two groups: time spent outdoors. “Before we did this study, we did not know that the main difference would be outdoors,” she admits. “We had about twenty questions on reading and writing; we only had one question on outdoors, and the most striking difference was the outdoor time.”

The children in Sydney were found to be outdoors for an average of almost 14 hours of every week, while those in Singapore averaged only 3 hours outdoors. Saw adds that more detailed studies have been unable to draw distinctions between any specific type of outdoor activity, from hard exercise to lounging under the sun. “This is not the inverse of near work,” she emphasizes. “It wasn’t because they were spending more time outdoors and less time on near work. We do not know exactly what activity protects from myopia. But we do know from the studies that the total time outdoors was protective.”

Clinical trials support outdoor time as an effective intervention. A pilot trial in Taiwan reported a 50% reduction in new cases of myopia by simply locking classroom doors during school recess, which prevented the children from staying indoors and working.^2^ And in a larger trial in Guangzhou, adding 45 minutes of structured time outdoors each day was associated with a 25% reduction in new cases of myopia.^29^ This study also included an information campaign targeted at parents.^30^

Morgan, who coauthored the Guangzhou study, says, “The epidemiology indicates that there is a dose–response relationship between time outdoors and protection, so the expectation is that if we can lift the amount of time outdoors up to closer to the Australian norm, then greater protection would be achieved.” He says children in Australia get outside an average of 4.5 hours per day, both through general activities and at school, compared with about 1.5 hours per day for children in Guangzhou and 2.8 hours for children in Taiwan.

## Taking Action

Singapore’s “Go outdoors and play” campaign speaks to a growing acceptance that outdoor light is protective. Taiwan, on the other hand, has adopted a pharmacological response—the growing use of atropine, an agent that paralyzes eye muscles and dilates the pupil. Proponents defend its use as a means of slowing down the progress of myopia after it has been diagnosed in children, but the long-term effects of this treatment remain unknown.^31^

For Chinese children, Morgan sees the education system as the real nemesis of eyesight, because urging parents to get their kids outside will do no good if schoolwork continues to take priority over health. “The choice is between encouraging people to spend more time outdoors and mandating more time outdoors through the school system,” Morgan says. “By and large Singapore has opted for persuasion, but all sorts of considerations suggest that making [outdoor time] part of the delivery of education may be more effective.”

In touting sunlight exposure as a preventive measure, Morgan acknowledges a major issue that must be confronted, “namely that increasing time outdoors also has the potential to promote skin cancer—an issue which I am acutely aware of, as an Australian.” (Australia and New Zealand have the world’s highest incidence and mortality rates of cutaneous melanoma.^32^) Here, he says, the mechanism becomes important. If protection is conferred by vitamin D, which is produced in the skin by ultraviolet light exposure—a hypothesis Mutti is pursuing^33^^,^^34^—then myopia prevention would be incompatible with skin protection. But Morgan points to findings in chicks indicating that both daylight and intense ultraviolet-free indoor light conferred a protective effect.^35^ This, he says, suggests myopia prevention depends on visible light acting through the eye.

Although significant progress has been made so far, the importance of further clarifying the relationship between myopia and the environment is clear. “Even if successful prevention becomes possible, East Asia will still be faced for close to the next one hundred years, with an adult population at high risk of developing pathological myopia,” Morgan says. “Further progress in our understanding of the natural history of pathological myopia is thus essential, and while there have been some promising developments in treatment, more effective treatments are still required.”
